# Remote sensing of aerosols over drylands: Challenges, uncertainties, and paths forward

**DOI:** 10.1126/sciadv.aec4247

**Published:** 2026-07-29

**Authors:** Cheng Chen, Pavel Litvinov, Oleg Dubovik, Thomas F. Eck, Elena S. Lind, Gerrit de Leeuw, Zhengqiang Li

**Affiliations:** ^1^ Anhui Institute of Optics and Fine Mechanics, Hefei Institutes of Physical Science, Chinese Academy of Sciences, Hefei 230031, China.; ^2^ GRASP-SAS, Remote Sensing Developments, F-59000 Lille, France.; ^3^ Anhui Province Key Laboratory of Optical Quantitative Remote Sensing, Hefei Institutes of Physical Science, Chinese Academy of Sciences, Hefei 230031, China.; ^4^ Univ. Lille, CNRS, UMR 8518–LOA–Laboratoire d’Optique Atmosphérique, F-59000 Lille, France.; ^5^ NASA Goddard Space Flight Center, Greenbelt, MD 20771, USA.; ^6^ GESTAR-II, University of Maryland Baltimore County, Baltimore, MD 21228, USA.; ^7^ State Environmental Protection Key Laboratory of Satellite Remote Sensing and Key Laboratory of Remote Sensing and Digital Earth, Aerospace Information Research Institute, Chinese Academy of Sciences, Beijing 100101, China.; ^8^ R&D Satellite Observations, Royal Netherlands Meteorological Institute (KNMI), 3730AE De Bilt, Netherlands.; ^9^ State Key Laboratory of Spatial Datum, College of Remote Sensing and Geoinformatics Engineering, Faculty of Geographical Science and Engineering, Henan University, Zhengzhou 450046, China.

## Abstract

Remote sensing of atmospheric aerosols has advanced substantially over recent decades, driven by progress in satellite instrumentation and the expansion of ground-based networks such as AERONET. While agreement between satellite aerosol optical depth (AOD) retrievals and AERONET reference data has improved, our analysis highlights a critical yet underrecognized imbalance in global validation frameworks. Specifically, AERONET sites are disproportionately concentrated in urban and vegetated regions, where fine-mode aerosols over dark surfaces favor retrieval accuracy, while drylands, dominated by coarse-mode aerosols over bright surfaces, are underrepresented by nearly a factor of two. This sampling bias introduces a systematic distortion in global validation outcomes. We show that nearly half of the global grid cells with elevated disagreement between MODIS and POLDER AOD products are located in drylands with angström exponent values below 0.75. In these areas, the mean top-of-atmosphere aerosol radiative cooling is weaker by 0.24 watts per square meter than in other regions and has an associated uncertainty of 22% higher. These findings highlight that, for improving estimation of global aerosol effects on climate, there is a need for a more stratified validation framework based on surface type and aerosol regime and an importance of continuing improving ground-based observations over drylands, with some network expansion if possible.

## Teaser

Dryland underrepresentation biases aerosol retrieval, limits algorithm performance, and increases radiative effect uncertainties.

## INTRODUCTION

Bare soil and desert, or arid and semiarid drylands, usually appear brown and slightly reddish on true color satellite top-of-atmosphere (TOA) RGB composed images and cover more than 35% of Earth’s land surface (*1*–*4*). Although these areas are largely inaccessible and primarily sources of natural desert dust (DD), climate change–induced crises, such as extreme weather and intensified drought, cannot be ignored in these regions (*5*–*8*). Satellite remote sensing is a unique method for obtaining the spatial distribution of atmospheric aerosols globally. However, the surface in bare soil and desert regions is generally bright and dominates the TOA satellite–measured signals, which complicates distinguishing and characterizing the contribution of the atmosphere to satellite observations (*9*–*11*). Ground-based remote sensing provides a vital means of accurate aerosol monitoring because the contribution of the surface reflectance to ground observations is minor. However, because of limited accessibility, maintaining instruments and conducting continuous observations over drylands are associated with substantial complications, making such observations particularly valuable. Thus, for the abovementioned reasons, both spaceborne satellite and ground-based remote sensing observations of atmospheric aerosols in most of these regions are limited overall and even insufficient (*12*, *13*).

The ground-based Aerosol Robotic Network (AERONET) is a federation of regional and national networks deployed in the 1990s by the collaboration of NASA with Laboratoire d’Optique Atmosphérique/University of Lille in the form of automatic stations for aerosol monitoring (*14*, *15*). On the basis of the well-established calibration scheme of sun-sky photometers, cloud screens (*14*, *16*), retrieval methods (*17*–*21*), and quality assurance procedures (*16*), AERONET delivers timely, unique, and high-quality aerosol products that have been considered as ground-truth in satellite remote sensing retrieval validation, chemical transport model evaluation, and long-term climatology analysis (*22*–*35*). Although AERONET has grown from the first few stations to more than 600 permanent stations today, by assuming that each AERONET station represents a 30 km–by–30 km area, it covers only ∼0.09% of the world surface area (510.1 million km^2^). Therefore, it is important that observations at AERONET stations provide sampling ideally representative of the global atmosphere over both ocean and land, including less accessible arid and semiarid zones, especially since these areas cover more than 35% of Earth’s land surface. However, it is not possible for AERONET (or any ground-based network) to provide the same representative sampling over all surface types on Earth, due to the constraints of logistic difficulties, operational cost, and inaccessibility in most remote regions, as well as political constraints.

Satellite remote sensing of aerosol over land, especially bare soil and desert regions, typically relies on predefined aerosol models and/or a priori information on surface reflectance. Therefore, AERONET observations are highly valuable not only for validating satellite observations but also for designing retrieval approaches. For example, aerosol models of size distributions and absorption from AERONET climatology are used to derive aerosol optical depth (AOD) retrievals from single-viewing spectral satellite images (e.g., MODIS and VIIRS) via many algorithms, such as the Deep Blue (DB) (*36*–*38*) and Multi-Angle Implementation of Atmospheric Correction (MAIAC) algorithms (*39*). In these retrievals, the AERONET climatological aerosol models fully or, to a significant degree, define the expected spectral dependence of AODs that relates to the type of the observed aerosol. Correspondingly, sufficient representativeness of AERONET observations everywhere and especially over arid and semiarid areas is very important for providing accurate satellite aerosol products. Moreover, recent successes in the development of advanced aerosol monitoring methods from observations by satellite multiangular polarimeters (MAPs) have demonstrated the possibility of reliably retrieving aerosol microphysical properties without using predetermined aerosol models (*40*–*50*). The recent global enhanced aerosol products generated from MAPs (*25*, *44*, *48*) made it possible to take a glance at the global spatial distribution of detailed aerosol microphysical properties, such as the aerosol size indicator angström exponent (AE) (*51*), including drylands and other remote areas.

Here, we benefitted from the enhanced aerosol dataset newly generated by the Generalized Retrieval of Atmosphere and Surface Properties (GRASP) algorithm applied to satellite measurements from the Polarization and Directionality of the Earth’s Reflectances (POLDER) multiangular polarimetric data (*25*, *40*, *49*). Specifically, we conducted a comparative analysis of the state-of-the-art POLDER/GRASP aerosol data, widely used MODIS aerosol products, and ground-based AERONET aerosol datasets obtained over the past two decades with the aim of revealing improvements over time in the agreement of satellite products with the AERONET ground-truth AOD. The analysis seems to show that apparent improvement in aerosol satellite retrieval (as shown in Results) is marginal at best and may not represent a true improvement at all. While global comparisons do indicate an increased overall agreement between satellite MODIS product and AERONET AOD. This improvement is normally attributed to maturing of retrieval approaches, while on the basis of the present analysis, it appears to be driven primarily by a growing volume of AERONET observations from regions that are more favorable for retrieval algorithms—namely, areas characterized by vegetated or urban surfaces and dominated by fine-mode aerosols. The increasing proportion of such retrieval-friendly data in validation datasets has positively biased overall statistical metrics of satellite performance. In contrast, a more targeted analysis reveals that substantial discrepancies persist over bare soil and desert regions, where coarse-mode nonspherical aerosols dominate and surface conditions are more challenging. These findings underscore the pressing need to enhance the accuracy of satellite aerosol retrievals over arid and semiarid regions. Certainly, the increase in ground-based measurements over drylands would be highly beneficial for achieving this objective. At the same time, it is evident that the density of AERONET ground-based sites over very sparsely populated drylands would hardly ever approach the density of sites over densely populated regions, since these sites require infrastructure and human maintenance.

## RESULTS

### Growing relative scarcity of ground-based observations in bare soil and desert regions

We examined the global coverage of ground-based AERONET Version 3 Level 2 direct Sun observations in 2008 and 2018, using satellite-derived aerosol and surface data to assess spatial representativeness. Specifically, we analyzed the global distribution of the aerosol AE from the POLDER/GRASP dataset, which is the first quantitative, global AE product derived from multiangular polarimetric satellite observations (see Materials and Methods). Surface characteristics were assessed using the MODIS/MAIAC Collection 6.1 daily surface normalized difference vegetation index (NDVI) datasets at 0.05° × 0.05° resolution for the same years. On the basis of NDVI values, surface types were categorized as desert and bare soil (0 < NDVI ≤ 0.2), transitional zones (0.2 < NDVI ≤ 0.4), vegetated (0.4 < NDVI ≤ 0.6), and densely vegetated (NDVI > 0.6) regions.

**Fig. 1.**
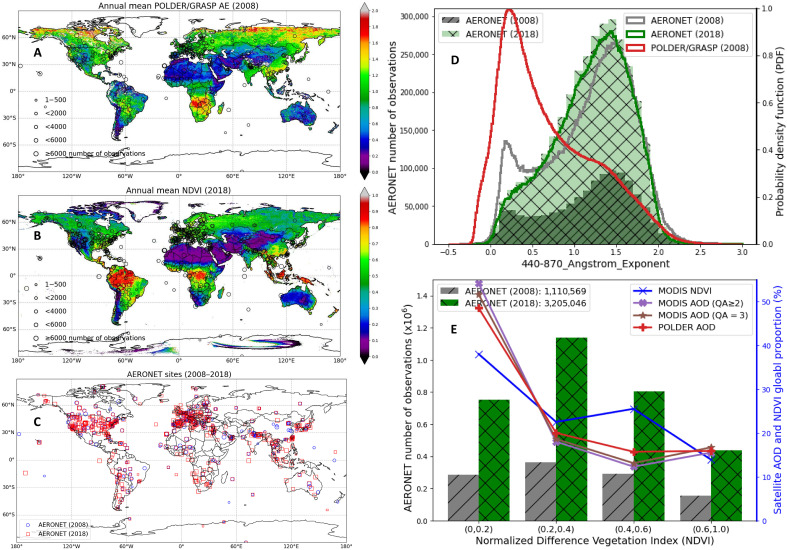
Spatial distributions of vegetation, aerosol properties, and AERONET observational coverage in 2008 and 2018. (**A**) Global distribution of annual mean aerosol AE from POLDER/GRASP in 2008. (**B**) Global distribution of the annual mean surface NDVI from MODIS/MAIAC in 2008. (**C**) Global distribution of AERONET sites in 2008 and 2018. In (A) to (C), the number of AERONET Version 3 Level 2 direct Sun observations at each site is overlaid. (**D**) Frequency of occurrence and probability density functions (PDFs) of AE from AERONET and POLDER in 2008 and 2018. (**E**) Occurrence frequency of AOD measurements from AERONET, MODIS, and POLDER across four land surface classes defined by NDVI: bare soil and desert (0 < NDVI ≤ 0.2), semiarid (0.2 < NDVI ≤ 0.4), vegetated (0.4 < NDVI ≤ 0.6), and densely vegetated (0.6 < NDVI ≤ 1), in 2008 and 2018. QA, quality assurance.

Figure 1 shows the global distribution of AERONET sites and the number of valid observations collected in 2008 and 2018, overlaid on maps of the POLDER/GRASP annual mean aerosol AE (Fig. 1A), MODIS/MAIAC annual mean NDVI maps (Fig. 1B), and the global distribution of AERONET sites in 2008 and 2018 (Fig. 1C). AE is an aerosol intrinsic property for which the annual mean POLDER/GRASP AE is calculated using the annual mean AODs at 443 and 865 nm. A clear spatial correspondence is observed between aerosol AE (Fig. 1A) and the surface NDVI (Fig. 1B), with low AE values, indicative of coarse-mode particles, primarily found in regions with low NDVI, such as deserts and bare soils. This pattern is consistent with the natural origin of coarse aerosols, particularly mineral dust, from sparsely vegetated or unvegetated drylands. In contrast, higher AE values, typically associated with fine-mode aerosols from anthropogenic sources, e.g., biomass burning and urban pollution, are predominantly observed in areas with moderate to high vegetation cover.

A notable trend over the decade between 2008 and 2018 is the substantial growth in AERONET network coverage (Fig. 1C). The number of active sites increased from 284 in 2008 to 497 in 2018, accompanied by a near threefold increase in the volume of high-quality Level 2 direct Sun observations, from ∼1.1 million to ∼3.2 million. Despite this expansion, a large proportion of the observational growth occurred in vegetated regions (NDVI > 0.2) (Fig. 1B), particularly populated regions across Europe, North America, as well as urban centers in developing countries. This highlights a persistent imbalance in ground-based aerosol monitoring, with drylands and sparsely vegetated regions remaining underrepresented.

To quantitatively assess the actual global coverage of different aerosol types and surface classes within AERONET observations, we used satellite remote sensing products from POLDER and MODIS. The AE was used to classify aerosol size modes, with AE ≥ 0.75 indicating fine-mode dominance and AE < 0.75 indicating coarse-mode dominance (*52*, *53*). On the basis of more than 57 million global monthly mean samples from POLDER/GRASP in 2008, used to mitigate satellite swath and cloud cover effects, the fine and coarse modes accounted for 49.0 and 51.0%, respectively. However, it is noted that the lower cloud fraction over dryland and desert regions still results in a higher number of retrievals over these regions compared to all others. This is partly why 49% of the land surface data were for coarse-mode retrievals, while 35% of the Earth surface is arid lands. In contrast, AERONET ground-based observations from 2008 (∼1.1 million measurements) were dominated by fine-mode aerosols (76.1%), with only 23.9% coarse-mode cases. By 2018, the proportion of coarse-mode dominated cases in AERONET further declined to 18.5% of ∼3.2 million total measurements (Fig. 1D), indicating a decreasing representation of coarse-mode aerosols in the ground-based reference dataset over time.

Figure 1E compares aerosol and surface occurrence frequencies of aerosol and surface properties observed by spaceborne satellites (POLDER and MODIS) and ground-based AERONET across four land cover types. Between 2008 and 2018, the number of AERONET observations over desert and bare soil regions (0 < NDVI ≤ 0.2) increased by a factor of 2.5; however, their relative fraction of total AERONET observations declined from 25.9 to 23.9%. In contrast, satellite AOD data from POLDER/GRASP and MODIS/AQUA reveal that more than 45% of global aerosol observations originated from desert and bare soil areas that is, at least, twice the fraction found in any other surface class, in part due to smaller cloud fraction over desert areas (*54*). This is consistent with MODIS/MAIAC surface NDVI products showing that ∼38% of global land pixels correspond to desert and bare soil surface, a lower fraction globally than the percentage of dust aerosol retrieval.

These discrepancies highlight an imbalance in the spatial distribution of ground-based AERONET sites, which do not proportionally represent all major Earth surface ecosystems. When combined, AERONET data underrepresent observations over desert and bare soil surfaces, as well as coarse-mode aerosol occurrences. Furthermore, this imbalance has become more pronounced over time due to the limited new site opportunities in most desert regions, underscoring the need for continued monitoring in drylands and sparsely vegetated regions to improve the representativeness of aerosol observations.

### Persistent inconsistency in satellite aerosol datasets over bright surfaces and its implications

Satellite remote sensing of coarse-mode aerosols over bright surfaces, such as bare soil and desert regions, remains particularly challenging. This difficulty arises from the need to accurately separate weak atmospheric signals from strong surface reflectance, as well as to account for the nonsphericity of coarse particles (*36*, *55*–*57*). For instance, the classical MODIS Dark Target (DT) algorithm is limited to densely vegetated areas with high NDVI to ensure reliable retrieval of aerosol loading (*22*, *58*, *59*). Meanwhile, MODIS pixels with 0 < NDVI ≤ 0.2 are processed using the DB algorithm (*36*–*38*), which relies on shorter wavelength (∼410 nm) where surface contributions are relatively small relative to longer-wave Visible, Near-Infrared, and Short-Wave Infrared (VIS-NIR-SWIR) channels. More advanced techniques, such as multiangular polarimetric observations, offer enhanced information content and are generally suitable for aerosol retrieval over land, including bare soil surfaces (*41*). In addition, recent algorithms such as GRASP and MAIAC incorporate the multipixel concept (*39*, *40*), leveraging the relatively low temporal variability of surface reflectance to decouple surface and atmospheric signals across all surface types. However, MAIAC relies on a priori AERONET regional aerosol climatology for MODIS application. Despite these advancements, substantial variability and high uncertainty persist among satellite aerosol products over bright surfaces, as demonstrated by Chen *et al.* (*25*, *60*), underscoring the need for improved methodologies and additional ground-based validation activities in bare soil and desert regions.

In this study, we validate AOD at 550 nm from POLDER and MODIS for the years 2008 and 2018 using all available AERONET Version 3 Level 2 direct Sun AOD observations. Specifically, the recent 2008 POLDER/GRASP Level 3 0.1° × 0.1° AOD (550 nm) product processed by the optimized Chemical Components approach (*44*, *48*) and the MODIS/AQUA DT and DB combined AOD (550 nm) from 2008 and 2018 were used. The satellite-ground collocation follows the method of Chen *et al.* (*25*): Satellite AOD values are averaged over a 3 × 3 pixels window centered on each AERONET site, while AERONET AODs are averaged within ±30 min of the satellite overpass time. To ensure data quality, only satellite land pixels with 100% land coverage are considered. For POLDER/GRASP, we retain pixels with a relative fitting residual less than the minimum residual of the pixel over a year plus 3%, and for MODIS, only retrievals with quality assurance flag ≥ 2 are included.

**Fig. 2.**
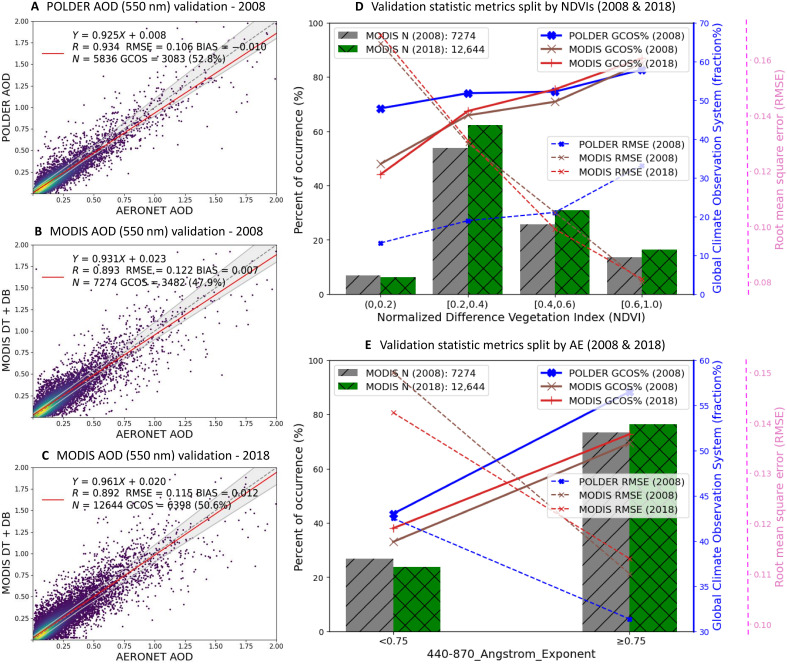
Validation of MODIS and POLDER AOD (550 nm) against AERONET in 2008 and 2018, with performance metrics split by the surface NDVI and aerosol AE. (**A**) Validation of POLDER/GRASP AOD against AERONET in 2008. (**B**) Validation of MODIS/AQUA DT and DB combined AOD against AERONET in 2008. (**C**) Validation of MODIS/AQUA DT + DB AOD against AERONET in 2018. (**D**) Validation performance metrics (e.g., RMSE and GCOS fraction) for POLDER/GRASP and MODIS/AQUA DT + DB AOD, split by surface NDVI classes (0 < NDVI ≤ 0.2, 0.2 < NDVI ≤ 0.4, 0.4 < NDVI ≤ 0.6, and 0.6 < NDVI ≤ 1) in 2008 and 2018. (**E**) Validation metrics for the same satellite products stratified by AE (<0.75 indicating coarse-mode dominance; ≥0.75 indicating fine-mode dominance) in 2008 and 2018.

Figure 2 presents the validation of the AOD (550 nm) from the MODIS/AQUA DT + DB merged product and the POLDER/GRASP retrievals against AERONET for the years 2008 and 2018. Specifically, comparisons are shown for POLDER/GRASP in 2008 (Fig. 2A), MODIS in 2008 (Fig. 2B), and MODIS in 2018 (Fig. 2C). Validation metrics, including Pearson’s linear correlation coefficient (*R*), linear regression slope and intercept, bias, root mean square error (RMSE), and the fraction of retrievals meeting the Global Climate Observation System (GCOS) requirement (see Materials and Methods), are reported for each case. Considering the ±0.01 uncertainty of the reference AERONET AOD (*52*), the AOD GCOS requirement used in this study is 0.04 or 10% AOD, whichever is greatest. In 2008, POLDER/GRASP retrievals (Fig. 2A) exhibited slightly better performance (*R* = 0.934, RMSE = 0.106, GCOS = 52.8%) compared to MODIS/AQUA DT + DB (Fig. 2B) (*R* = 0.893, RMSE = 0.122, GCOS = 47.9%). A comparison of MODIS products between 2008 (Fig. 2B) and 2018 (Fig. 2C) indicates a modest improvement over time, with RMSE reduced from 0.122 to 0.115 and GCOS fulfillment increasing from 47.9 to 50.6%. In addition, the number of collocated points increased by ∼74%, from 7274 in 2008 to 12,644 in 2018, due to the rapid growth and expansion of AERONET.

To better understand the apparent improvement in AOD validation metrics over time, we split the results by surface vegetation (NDVI, Fig. 2D) and aerosol type (AE, Fig. 2E). The analysis reveals that the enhanced agreement between satellite and AERONET observations is primarily driven by changes in the coverage evolution of AERONET reference dataset, rather than uniform improvements in retrieval accuracy. In particular, a disproportionate number of new AERONET sites was added in regions these satellite retrievals are relatively well constrained, typically vegetated areas with higher NDVI values, while coverage over drylands such as desert and bare soil regions (0 < NDVI ≤ 0.2) remained limited. Only 5.3% (284 of 5370) of the increase in collocated points between 2008 and 2018 occurred in these bare soil and desert areas, where MODIS AOD performance was consistently poor (RMSE > 0.16; GCOS fulfillment fraction < 33%). In contrast, in areas with NDVI > 0.2, RMSE remained below 0.13, and GCOS fraction exceeded 45%. The multiangular polarimetric POLDER/GRASP retrievals were less sensitive to a surface type, with RMSEs varying from 0.09 to 0.12 and GCOS fractions from 47.9% (0 < NDVI ≤ 0.2) to 51 to 58% (NDVI > 0.2) (Fig. 2D). Similarly, aerosol type played a role in the apparent improvement. Only 19.6% (1052 of 5370) of the new collocations involved coarse-mode aerosol conditions (AE < 0.75), while most additions occurred in fine-mode dominated regimes (AE ≥ 0.75). Both MODIS and POLDER/GRASP products showed better performance for fine-mode aerosols. For instance, POLDER/GRASP met the GCOS requirement in 43.0% of the coarse-mode cases versus 56.5% for fine-mode cases, with RMSEs 0.121 and 0.101, respectively (Fig. 2E). These findings suggest that the observed improvement in satellite-AERONET agreement over time is largely a result of biased sampling toward retrieval favorable conditions, specifically vegetated surfaces and fine-mode aerosols. To advance satellite aerosol monitoring globally, greater emphasis must be placed on validating and improving retrievals over bright surfaces and in coarse-mode aerosol environments.

**Fig. 3.**
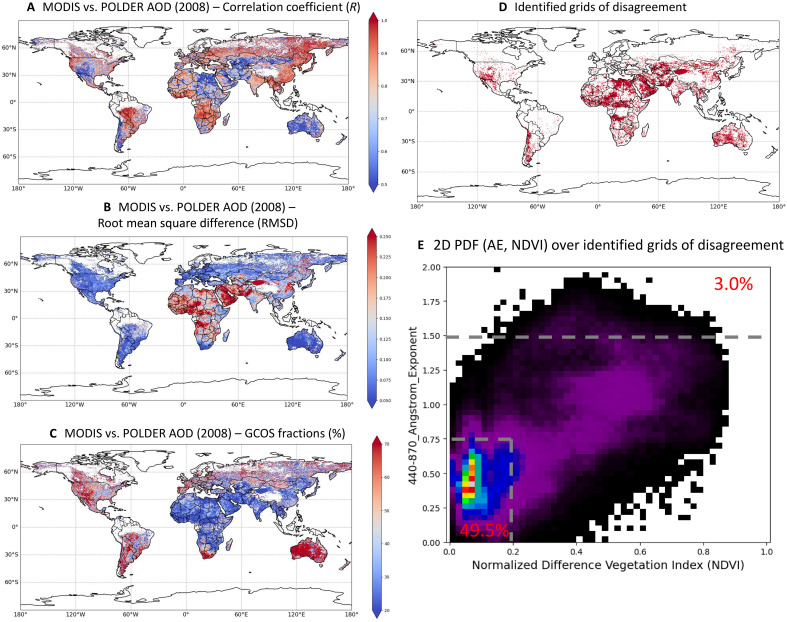
Spatial patterns of agreement between MODIS and POLDER AOD retrievals in 2008 and identification of regions of disagreement. (**A**) Pixel-level correlation coefficient (*R*) between MODIS and POLDER AOD (550 nm). (**B**) Pixel-level RMSD between the MODIS and POLDER AOD datasets. (**C**) Fraction of collocated pixels meeting the GCOS AOD accuracy requirement (GCOS%). (**D**) Geographic distribution of grid cells with consistently poor agreement across all three metrics (low *R*, high RMSD, and low GCOS%), indicating areas of disagreement. (**E**) Joint two-dimensional (2D) PDF of aerosol AE and surface NDVI for the grids identified in (D), illustrating the typical aerosol and surface characteristics associated with these low-consistency regions.

Both POLDER/PARASOL and MODIS/AQUA operated within NASA’s Earth Observing System (EOS) A-Train afternoon constellation in 2008, acquiring observations within minutes of each other. This orbital alignment provides an ideal basis for direct grid-to-grid comparison of satellite AOD retrievals. We conducted a global intercomparison of MODIS and POLDER AOD (550 nm) at 0.1° × 0.1° resolution for 2008, evaluating three statistical metrics per grid cell: Pearson’s correlation coefficient (*R*), root mean square difference (RMSD), and the fraction of pixels meeting the GCOS AOD accuracy requirement (GCOS; see Materials and Methods), the percentage of collocated MODIS AOD retrievals whose differences relative to POLDER/GRASP AOD fall within the GCOS uncertainty requirements for satellite aerosol products. In addition, all statistical metrics are calculated only for grid cells that contain at least 10 valid POLDER-MODIS collocated retrievals during the year 2008, ensuring sufficient sampling for robust statistical evaluation. The spatial distributions of these metrics are shown in Fig. 3 (A to C).

To identify regions with poor agreement between the two satellite products, we defined thresholds based on deviations from the global mean (±1 SD, σ). Globally, the mean and SD were *R* = 0.71 ± 0.24, RMSD = 0.140 ± 0.097, and GCOS% = 38.0 ± 22.1%. Accordingly, grid cells were flagged as inconsistent if *R* < 0.47, RMSD > 0.237, or GCOS% < 15.9%. The resulting set of poorly consistent grids (grids of disagreement) is shown in Fig. 3D. Further analysis of the surface and aerosol characteristics within these grids, based on the joint two-dimensional probability density function of NDVI and AE, is shown in Fig. 3E. A majority (49.5%) of these low-consistency regions are associated with drylands (0 < NDVI ≤ 0.2) and coarse-mode dominated aerosol conditions (AE < 0.75). In contrast, only 3% of the grids of disagreement correspond to strongly fine-mode dominated cases (AE > 1.5). As detailed in Materials and Methods, the comparison between POLDER and MODIS AOD over the ocean shows a high level of consistency (*R* = 0.952, RMSD = 0.044, GCOS = 78.1%). These findings reinforce the conclusion that discrepancies between satellite AOD products are most pronounced over drylands with coarse-mode aerosols, highlighting the need for improved retrieval strategies and enhanced validation in such environments.

To assess a presence of potential implications of satellite aerosol retrieval inconsistencies on climate forcing estimates, we examined the TOA clear-sky direct radiative effect (DRE) of speciated aerosols in regions of poor and good agreement between POLDER and MODIS AOD (hereafter “grids of disagreement” and “grids of agreement”; see Fig. 3D). The global clear-sky aerosol DRE is simulated with the GEOS-Chem model coupled with the Rapid Radiative Transfer Model for General Circulation Models Applications (RRTMG) (*61*) constrained by multiangular polarimetric POLDER/GRASP observations (*62*). Details about the simulation of clear-sky DRE are presented in Materials and Methods. The global aerosol DRE was evaluated separately for these two categories (grids of disagreement and grids of agreement), with results summarized in Table 1.

**Table 1.** Intercomparison of TOA clear-sky DRE by aerosol species over regions with poor and good satellite AOD. Mean TOA clear-sky DRE (watts per square meter) associated with five major aerosol species, DD, organic matter (OM), black carbon (BC), sulfate-nitrate-ammonium (SNA), and sea salt (SS), is compared between grids of agreement and disagreement between MODIS and POLDER AOD retrievals (grids of disagreement as identified in Fig. 3D) and the remaining land areas with good agreement (grids of agreement). Uncertainties represent one SD (±1σ) of the DRE estimates across each region.

	TOA clear-sky DRE (W/m^2^)	
	Grids of agreement	Grids of disagreement—identified in Fig. 3D
All aerosols	−1.73 (1.8)	−1.49 (2.2)
DD	−0.38 (0.9)	−0.48 (1.2)
OM	−0.66 (0.7)	−0.71 (1.0)
BC	1.39 (1.2)	1.53 (1.3)
SNA	−1.50 (1.5)	−1.22 (1.5)
SS	−0.20 (0.2)	−0.22 (0.3)

Both regions exhibited net aerosol–induced cooling at the TOA. However, the mean cooling over grids of disagreement (−1.49 W/m^2^) was 0.24 W/m^2^ weaker than over grids of agreement (−1.73 W/m^2^). This reduced cooling is primarily attributed to higher surface reflectance in arid and semiarid regions, which enhances the warming contribution of absorbing aerosols, particularly iron oxides in mineral dust and black carbon (BC). Uncertainties in the DRE estimates were systematically larger over grids of disagreement. DRE uncertainty ($\sigma_{\text{DRE}}$, see in Materials and Methods) is estimated by perturbing aerosol emissions using the MAP-constrained absorbing aerosol emission database (MACE) top-down emission inventory derived from POLDER/GRASP observations and quantifying the resulting spread in GEOS-Chem RRTMG coupled radiative flux calculations (*61*, *62*). The total aerosol DRE uncertainty (1σ) was ∼22% higher in these regions (2.2 W/m^2^) than in grids of agreement (1.8 W/m^2^). Similar trends were observed across individual aerosol species. Although this analysis does not explicitly propagate aerosol optical property uncertainties into radiative effects, it indirectly accounts for them via satellite-constrained emission variability. These results indicate that regions exhibiting larger inconsistencies among satellite aerosol products also tend to exhibit larger uncertainties in aerosol radiative effects. This spatial association highlights the importance of improving aerosol retrieval performance and observational constraints over drylands, particularly in coarse-mode aerosol regimes, to better constrain regional and global aerosol–climate interactions.

Furthermore, the rapid adoption of artificial intelligence (AI) in satellite aerosol retrievals relies increasingly on AERONET observations as ground-truth training data (*63*–*67*). In this context, the representativeness of training samples across diverse land surface and aerosol regimes is critical. Because the AERONET network is unevenly distributed and sparse over dryland regions and coarse-mode dominated aerosol environments, AI models trained without accounting for this imbalance may exhibit degraded performance in precisely these regions. This limitation highlights the need to explicitly consider observational sampling representativeness when developing and applying AI-based aerosol retrieval algorithms, particularly for large-scale climate-relevant applications.

### Toward robust validation of satellite aerosol products using ground-based measurements

Given the logistical challenges of expanding ground-based aerosol observations in desert regions (*52*, *68*–*70*) and the substantial value already contained within the existing AERONET dataset, we propose practical strategies to strengthen the validation of satellite aerosol products. First, increased weighting should be assigned to AERONET sites located in arid and semiarid regions, where satellite retrievals are known to be less reliable. Second, statistical evaluation metrics should be normalized with respect to cases that are representative of these challenging conditions, particularly those dominated by coarse-mode aerosols in drylands. These adjustments can improve the robustness of validation efforts and help ensure that global performance assessments better reflect retrieval skill over difficult but climatically important surfaces.

**Fig. 4.**
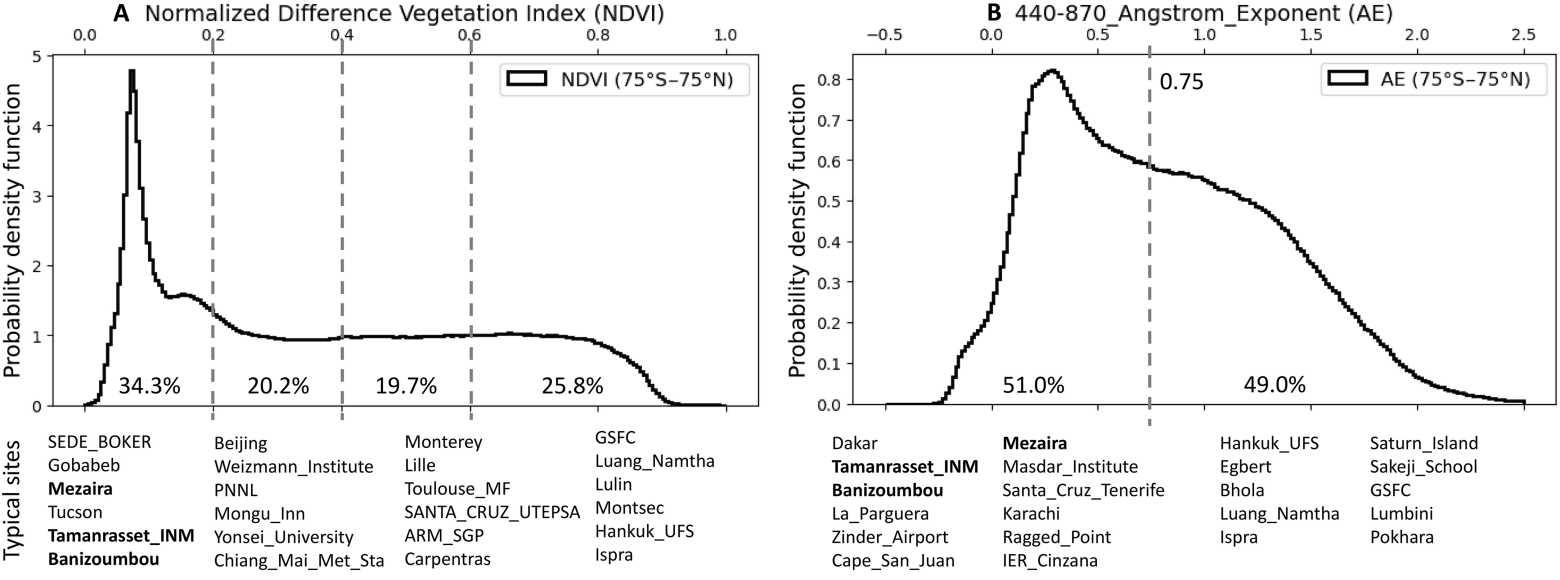
The global (75°S to 75°N) distribution of surface vegetation and aerosol type characteristics based on satellite (MODIS and POLDER) observations. (**A**) Probability density distribution of surface NDVI across four land surface classes (0 < NDVI ≤ 0.2, 0.2 < NDVI ≤ 0.4, 0.4 < NDVI ≤ 0.6, and 0.6 < NDVI ≤ 1), based on global satellite observations from 75°S to 75°N over a 1-year period. The frequency of occurrence within each NDVI class is shown, along with representative AERONET sites. (**B**) Probability density of the aerosol AE categorized into coarse-mode aerosol (AE < 0.75) and fine-mode aerosol (AE ≥ 0.75) regimes. Occurrence frequencies are presented with selected typical AERONET sites associated with each aerosol type.

Figure 4 illustrates the global probability distribution of satellite observed NDVI (MODIS/MAIAC) and AE (POLDER/GRASP) over a 1-year period between 75°S and 75°N. Statistical analysis of occurrence frequency (Fig. 4A) shows that 34.3% of the observations fall within arid and semiarid regions (0 < NDVI ≤ 0.2), 20.2% over mixture of bare soil and vegetated transitional surfaces (0.2 < NDVI ≤ 0.4), 19.7% over moderately vegetated areas (0.4 < NDVI ≤ 0.6), and 25.8% over densely vegetated regions (0.6 < NDVI ≤ 1). To ensure a more representative validation of satellite aerosol products across these diverse surface types, we propose a linear normalization of the validation statistics, e.g., RMSE and GCOS fraction, according to the global NDVI and AE occurrence distribution (see Materials and Methods). Applying this normalization to the NDVI stratified validation results in Fig. 2D reveals that the MODIS AOD performance in 2008 (RMSE = 0.125, GCOS% = 46.0%) and 2018 (RMSE = 0.125, GCOS% = 46.2%) is statistically comparable, contradicting the apparent improvement inferred from unnormalized metrics. In contrast, the POLDER/GRASP multiangular polarimetric retrievals exhibit greater consistency across surface types, with normalized validation results yielding an RMSE of 0.105 and GCOS compliance of 52.1%. A similar normalization can be applied on the basis of aerosol size regimes, using the global frequencies of coarse-mode (AE < 0.75, 51.0%) and fine-mode (AE ≥ 0.75, 49.0%) dominated cases, as shown in Fig. 4B. When the validation statistics stratified by AE in Fig. 2E are normalized accordingly, the MODIS AOD performance remains comparable between 2008 (RMSE = 0.130, GCOS% = 45.2%) and 2018 (RMSE = 0.128, GCOS = 46.5%). By contrast, the POLDER/GRASP retrievals demonstrate better performance, with AE-normalized validation yielding an RMSE of 0.111 and a GCOS compliance rate of 49.6%.

Furthermore, representative AERONET sites within four NDVI classes and two AE regimes have been identified on the basis of their extensive provision of Level 2 AOD observations in 2018. We highlight the importance of prioritizing sites in arid and semiarid regions, such as SEDE_BOKER, Mezaira, Tamanrasset_INM, Banizoumbou, etc., in future algorithm development and validation efforts, given their critical role in constraining coarse-mode aerosol retrievals over bright surfaces.

## DISCUSSION

Over the past three decades, sustained efforts to develop long-term global aerosol observation systems have led to a rapid expansion of remote sensing data from both ground-based and satellite platforms. Notably, the agreement between satellite-derived AOD products and ground-based AERONET reference measurements has improved significantly in the past decade, as reflected by a higher fraction of satellite retrievals meeting the GCOS requirements for AOD. Despite this progress, our comprehensive analysis of satellite (POLDER/GRASP and MODIS DT + DB) and ground-based (AERONET) aerosol datasets highlights two critical aspects that warrant further attention from the aerosol remote sensing community.

1) Over the past decade, the number of ground-based AERONET direct Sun AOD observations has increased by at least a factor of three. However, a growing proportion of new stations is located in urban areas that are either vegetated or partially vegetated (e.g., NDVI > 0.2) and are typically dominated by fine-mode aerosols. In contrast, the fraction of AERONET observations in bare soil and desert drylands (∼24%) remains significantly lower than the global surface coverage of such regions (∼35%). Moreover, the fraction in the bare soil and desert regions even decreased in the AERONET dataset due to the logistical difficulties establishing new sites in desert regions. Correspondingly, the proportion of coarse-mode dominated cases (AE < 0.75) in the AERONET declined from 23.9% in 2008 to 18.5% in 2018. These values fall well below the global coarse-mode dominant fraction of ∼51.0% estimated by the multi-angular polarimetric remote sensing POLDER/GRASP product. This imbalance suggests that coarse-mode aerosols over bright surfaces are increasingly underrepresented in ground-based reference datasets, a limitation that must be acknowledged in satellite validation efforts.

2) While recent satellite AOD products demonstrate good overall agreement with ground-based reference AERONET (*R*, ∼0.9; RMSE, ∼0.11; and GCOS fraction, ∼50%), such metrics may overstate global performance if validation samples underrepresent key surface types and aerosol regimes. In particular, our 1-year 0.1° × 0.1° grid-to-grid intercomparison of MODIS and POLDER AODs identified regions of poor agreement (defined as any grid falling below global mean ± 1σ in at least one of the metrics: *R*, RMSD, or GCOS fulfillment fraction). Approximately 50% of these grids of disagreement are located in bare soil and desert regions (0 < NDVI ≤ 0.2) and dominated by coarse-mode particles (AE < 0.75), while only 3% are associated with strong fine-mode dominance (AE > 1.5). Moreover, the TOA clear-sky aerosol DRE (cooling effect) in these grids of disagreement between POLDER and MODIS AOD is ∼0.24 W/m^2^ weaker (less cooling) than in well-agreeing grids, and the associated DRE uncertainty is ∼22% higher.

Last, these findings underscore the critical need for continued observational coverage in arid and semiarid regions. Maintaining and, if possible, expanding ground-based aerosol monitoring in bright surface and coarse-mode aerosol regimes is essential for enhancing the consistency of satellite aerosol products and for reducing uncertainty in global aerosol radiative effect assessments.

## MATERIALS AND METHODS

### Global distribution of aerosol size regimes as characterized by the AE from POLDER/GRASP retrievals

The aerosol AE is a measure of the spectral dependency of AOD as a proxy for aerosol particle size (*51*). Given AOD (τ) at two wavelengths (λ), the AE (α) is derived using the following relationship

$$\alpha =-\text{ln}\left(\frac{\tau_{\lambda_{1}}}{\tau_{\lambda_{2}}}\right)/\text{ln}\left(\frac{\lambda_{1}}{\lambda_{2}}\right)$$ (1)

The AE provides a proxy for aerosol particle size with values below 0.75 typically indicating coarse-mode aerosols such as DD and values above 0.75 suggesting fine-mode particles including sulfate, BC, and organic matter (OM) (*52*, *68*). Although AE is widely used in aerosol classification (*71*), its retrieval from spaceborne observations remains challenging due to the high sensitivity to small errors in multiwavelength AOD. As a result, ground-based sun-photometer networks, such as AERONET, SKYNET, SONET, and CARSNET (*14*, *72*–*74*), remain the principal sources for reliable AE measurements, albeit with limited spatial coverage. The multiangular polarimetric aerosol product from POLDER, generated by the GRASP algorithm, offers the first global coverage aerosol AE (440 to 870 nm) with demonstrated high accuracy (*25*, *44*, *75*). In this study, we used the 2008 POLDER/GRASP AE (440 to 870 nm) product to characterize the global distribution of aerosol size (Fig. 1A) and evaluated its consistency with collocated AE derived from AERONET observations.

Figure S1 presents the validation of the POLDER/GRASP Level 3 AE (440 to 870 nm) at 0.1° × 0.1° resolution against AERONET Version 3 Level 2 data. Following the collocation protocol of Chen *et al.* (*25*), satellite data were spatially averaged over a 3 × 3 pixel window centered on each AERONET site, while AERONET direct Sun AE observations were averaged within ±30 min of the satellite overpass. Note that the averaged AE is calculated using the mean AODs at corresponding wavelengths. Only land pixels (100% land fraction) with high-quality retrieval (relative residual < minimum pixel residual over a year + 3%) were included. To evaluate the stability of POLDER/GRASP AE retrievals, validation was performed for both all AOD conditions (fig. S1A) and for cases where POLDER AOD (550 nm) exceeded 0.1 (fig. S1B). For comparison, the same validation strategy was applied to MODIS/AQUA DT and DB AE products (fig. S2). POLDER/GRASP demonstrates robust satellite-based fine/coarse mode discrimination with quantitatively accurate AE retrievals. Correlation coefficients (*R*) range from ∼0.7 to 0.75, RMSE falls between 0.4 and 0.5, and more than 70% of the retrievals satisfy the ±0.5 AE requirement across all AOD conditions. In contrast, MODIS products exhibit discretized AE values, a result of predefined spectral AOD dependencies based on climatological aerosol models from AERONET, limiting their flexibility in characterizing aerosol variability.

### Statistical parameters for validation and intercomparison

To evaluate the validation performance for AOD and AE, we used several statistical metrics in this study, including Pearson’s linear correlation coefficient (*R*), RMSE, the fraction meeting the GCOS requirement for AOD, and the formulated optimal and target requirements for AE

$$R=\frac{\sum \left(O_{i,\text{satellite}}-\bar{O_{\text{satellite}}}\right)\left(O_{i,\text{AERONET}}-\bar{O_{\text{AERONET}}}\right)}{\sqrt{\sum {\left(O_{i,\text{satellite}}-\bar{O_{\text{satellite}}}\right)}^{2}\sum {\left(O_{i,\text{AERONET}}-\bar{O_{\text{AERONET}}}\right)}^{2}}}$$ (2)

$$\text{RMSE}=\sqrt{\frac{\sum_{i=1}^{N}{\left(O_{i,\text{satellite}}-O_{i,\text{AERONET}}\right)}^{2}}{N}}$$ (3)

$$\text{BIAS}=\frac{1}{N}\sum_{i=1}^{N}\left(O_{i,\text{satellite}}-O_{i,\text{AERONET}}\right)$$ (4)

GCOS=max(0.04 or 10%AOD) (5)

$${\text{Optimal}}_{AE}=\pm 0.3$$ (6)

$${\text{Target}}_{AE}=\pm 0.5$$ (7)

where N is the number of satellite-AERONET matched data points i; $O_{\text{satellite}}$ represents the satellite observation; $O_{\text{AERONET}}$ represents the AERONET reference observation; and $\bar{O_{\text{satellite}}}$ and $\bar{O_{\text{AERONET}}}$ are the mean values for the satellite and AERONET data, respectively. Note that AOD GCOS requirement is also used in the POLDER and MODIS intercomparison as a consistency metric, defined as the percentage of collocated MODIS AOD retrievals whose differences relative to POLDER AOD fall within the GCOS uncertainty requirements for satellite aerosol products.

### Land cover and aerosol size–dependent normalization of validation performance

Validation results for POLDER/GRASP and MODIS/AQUA DT + DB AOD (550 nm), including RMSE and GCOS fulfillment fraction, are summarized in tables S1 and S2 across four land surface vegetation classes (0 < NDVI ≤ 0.2, 0.2 < NDVI ≤ 0.4, 0.4 < NDVI ≤ 0.6, and 0.6 < NDVI ≤ 1) and two aerosol size regimes (coarse mode: AE < 0.75; fine mode: AE ≥ 0.75), respectively. Normalization factors for each surface class and aerosol regime (Fig. 4) are also provided in the corresponding tables, with normalized metrics reported in brackets.

### Comparing POLDER and MODIS AOD retrievals over ocean surfaces

Although ground-based aerosol observations over ocean surfaces are sparse, as in desert regions, the relatively rich information content of satellite retrievals over dark ocean surfaces supports stronger consistency between satellite AOD products. Figure S3 illustrates the spatial distribution of AE (440 to 870 nm) and the intercomparison of ocean AOD (550 nm) between POLDER/GRASP and MODIS/AQUA DT retrievals, stratified by aerosol size regime. Globally, over ocean, 40.4% of cases are coarse-mode dominant (AE < 0.75) and 59.6% fine-mode dominant (AE ≥ 0.75). However, the coarse-mode particles over ocean are often sea salt (SS) which typically are spherical in shape and also at low AOD levels. We conducted a global intercomparison of MODIS and POLDER AOD (550 nm) at 0.1° × 0.1° resolution for 2008 over ocean. On the basis of more than 19 million ocean pixels, the correlation coefficient between POLDER and MODIS is 0.952, with an RMSD of 0.044, and 78.1% of pixels meet the AOD GCOS requirement, indicating a high level of consistency, in contrast to statistics over land (*R* = 0.710, RMSD = 0.140, GCOS fraction = 38.0%). When split by aerosol size regime, the agreement remains robust (coarse mode: *R* = 0.969, GCOS fraction = 79.9%; fine mode: *R* = 0.941, GCOS fraction = 77.3%). Although independent verification data over the ocean are limited, the enhanced atmospheric signal over the dark ocean surface reduces retrieval uncertainty and strengthens product consistency. Overall, in contrast to bright desert surfaces where remote sensing remains more challenging, these results highlight the need to expand ground-based observations to improve inversion algorithms and the consistency of satellite aerosol products.

### GEOS-Chem simulation of clear-sky DRE constrained by POLDER/GRASP

We computed the clear-sky DRE using the GEOS-Chem v11-01 chemical transport model (https://geoschem.github.io/index.html). The model includes five externally mixed aerosol components: BC, OM, DD, sulfate-nitrate-ammonium (SNA), and SS. Simulations were performed at a horizontal resolution of 2° × 2.5° and were driven by GEOS-FP meteorological fields. To better constrain absorbing aerosol sources, we used the top-down MAP retrieval–based MACE daily emission dataset derived from POLDER/GRASP observations (*76*, *77*). These MACE emissions were used for BC, OM, and DD, while SNA and SS were simulated following the parameterizations described in (*78*, *79*). A fast radiative transfer module (RRTMG), integrated within GEOS-Chem, was applied to compute shortwave and longwave aerosol radiative effects based on the simulated speciated aerosol optical properties (*61*). To obtain the clear-sky DRE, two radiative transfer simulations are performed: one including all aerosol species and another with aerosol extinction set to zero. The DRE is diagnosed as the difference in net radiative fluxes between these two simulations

$${\text{DRE}}^{\text{TOA}}=F_{\text{net},\;\text{aerosol}}^{\text{TOA}}-F_{\text{net},\;\text{no}\;\text{aerosol}}^{\text{TOA}}$$ (8)

where net flux is defined as the difference between downward and upward radiative fluxes. In this study, we consider DRE at TOA under clear-sky conditions with cloud radiative effects removed.

The use of MACE daily emissions enables improved simulation of daily spectral AOD and aerosol absorption optical depth (AAOD), therefore better constrained estimation of clear-sky DRE for the period 2006–2011 used in this study. DRE uncertainty ($\sigma_{\text{DRE}}$) is estimated by perturbing aerosol emissions using the MACE top-down emission inventory derived from POLDER/GRASP observations and quantifying the resulting spread in GC-RT (GEOS-Chem RRTMG) radiative flux calculations

$$\sigma_{\text{DRE}}=\sqrt{\frac{1}{N}\sum_{i=1}^{N}{\left({\text{DRE}}_{i}-\bar{\text{DRE}}\right)}^{2}}$$ (9)

where ${\text{DRE}}_{i}$ is the DRE from the *i*th perturbed emission simulation, $\bar{\text{DRE}}$ is the mean DRE across all perturbation cases, and N is the total number of perturbed simulations. This approach captures the sensitivity of aerosol radiative effects to uncertainties in emission strength and spatial distribution, particularly for absorbing aerosols such as BC, OM, and DD, which strongly influence radiative effects. The GEOS-Chem–simulated speciated daily AOD and AAOD, and clear-sky DRE are publicly available at https://doi.org/10.5281/zenodo.6348890 (last accessed: 16 December 2025). Additional details on the simulation framework, including aerosol optical properties and emission treatments, are provided in (*62*).
