# Supplementary material for: Remote sensing of aerosols over drylands: Challenges, uncertainties, and paths forward
Source: Sci Adv. 2026 Jul 29;12(31):eaec4247. doi: 10.1126/sciadv.aec4247 (PMC13418731; doi:10.1126/sciadv.aec4247)
Supplement: Supplementary file 1 — Figs. S1 to S3 Tables S1 and S2 [file sciadv.aec4247_sm.pdf]

Science Advances
AAAS

Supplementary Materials for

Remote sensing of aerosols over drylands: Challenges, uncertainties, and paths forward

Cheng Chen *et al.*

Corresponding author: Cheng Chen, cheng.chen@aiofm.ac.cn; Oleg Dubovik, oleg.dubovik@univ-lille.fr; Zhengqiang Li, lizq@radi.ac.cn

Sci. Adv. **12**, eaec4247 (2026)
DOI: 10.1126/sciadv.aec4247

This PDF file includes:

Figs. S1 to S3
Tables S1 and S2

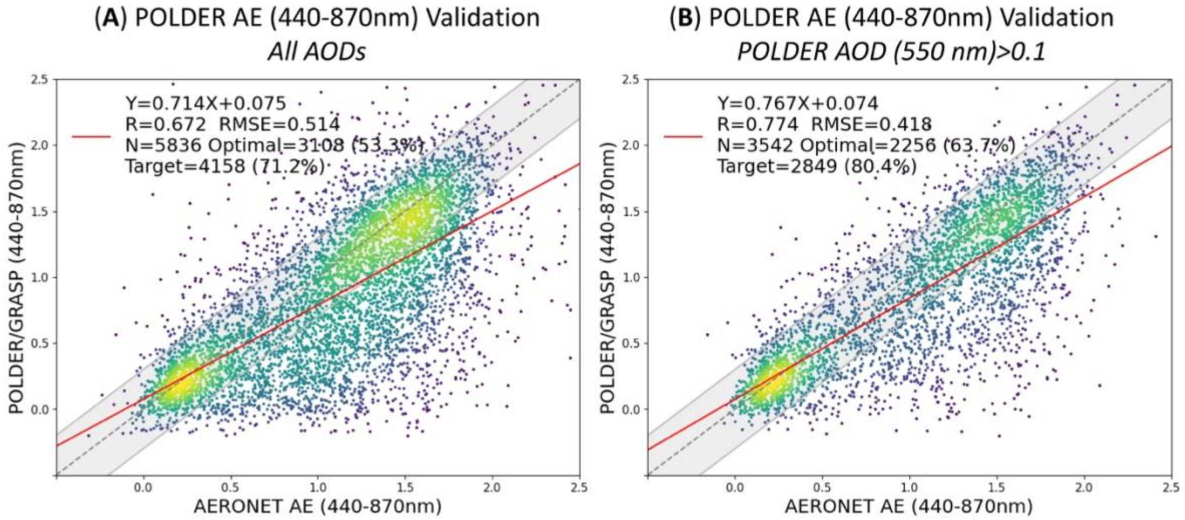

Fig. S1. Validation of POLDER/GRASP Ångström exponent against AERONET. Comparison of POLDER/GRASP Ångström exponent (AE, 440-870 nm) with AERONET Level 2 AE (440-870 nm) under (**A**) all aerosol optical depth (AOD) conditions and (**B**) moderate-to-high AOD conditions with POLDER AOD (550 nm) > 0.1.

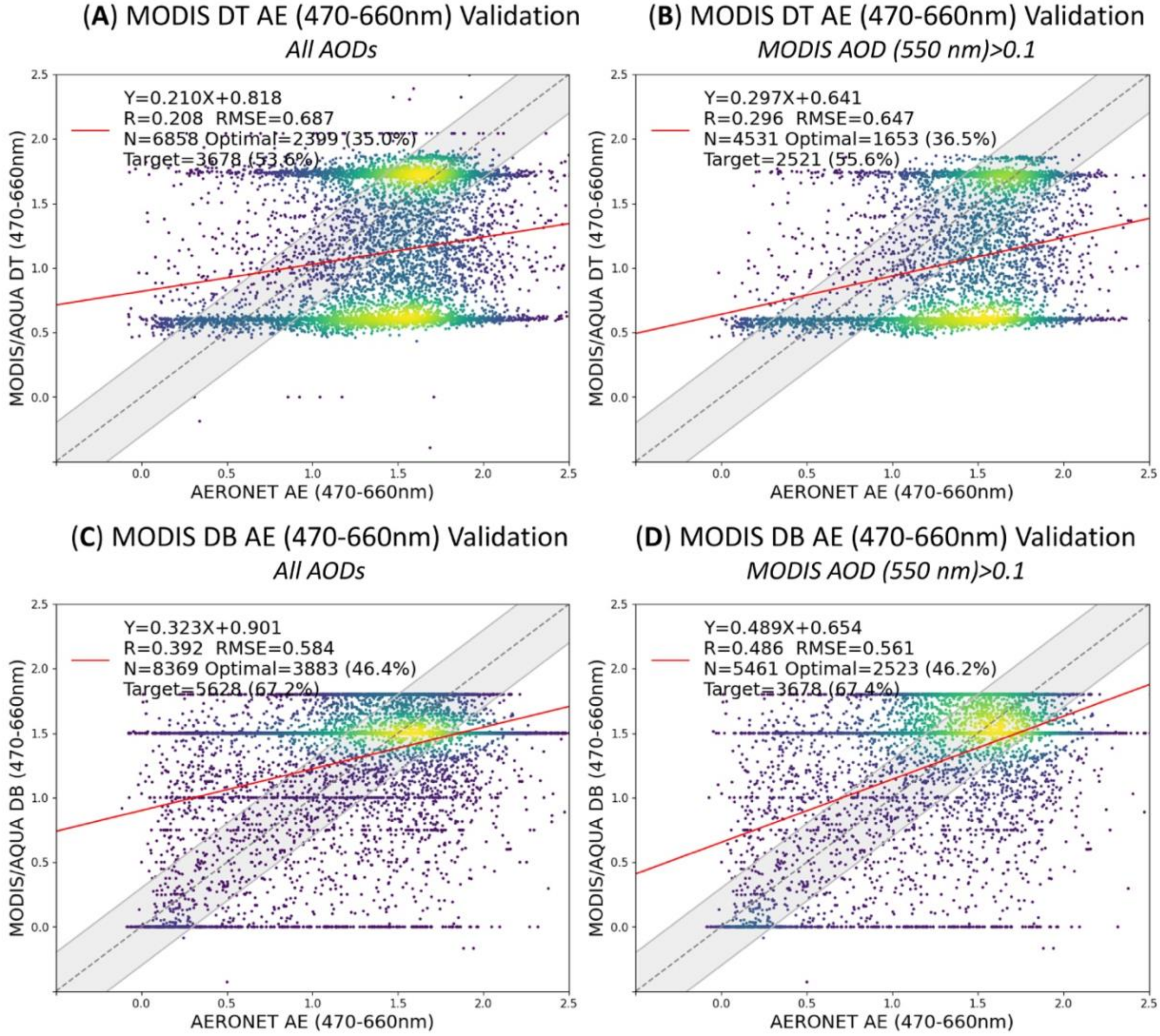

Fig. S2. Validation of MODIS/Aqua Dark Target and Deep Blue Ångström exponent against AERONET. Comparison of MODIS/Aqua Dark Target (DT) and Deep Blue (DB) Ångström exponent (AE, 470-660 nm) with AERONET Level 2 AE (470-660 nm) in 2008 for (**A**) MODIS/Aqua DT under all aerosol optical depth (AOD) conditions; (**B**) MODIS/Aqua DT under moderate-to-high AOD conditions with MODIS AOD (550 nm) > 0.1; (**C**) MODIS/Aqua DB under all AOD conditions; and (**D**) MODIS/Aqua DB under moderate-to-high AOD conditions with MODIS AOD (550 nm) > 0.1.

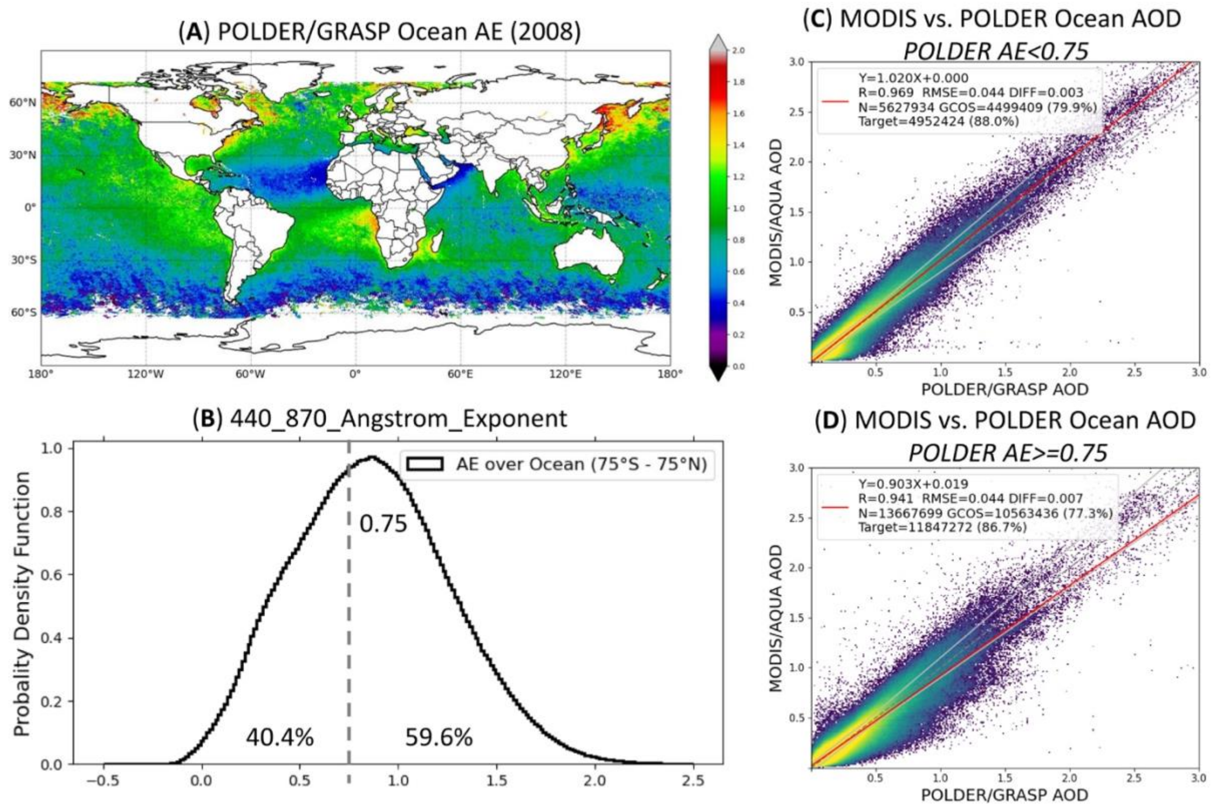

Fig. S3. Spatial patterns of aerosol Ångström exponent and intercomparison of POLDER/GRASP and MODIS/AQUA ocean AOD. (**A**) Spatial distribution of POLDER/GRASP Ångström exponent (AE, 440-870 nm) over ocean in 2008; (**B**) Probability density distribution of AE over ocean, categorized into coarse-mode aerosol (AE<0.75) and fine-mode aerosol (AE≥ 0.75) regimes, based on satellite observations between 75°S and 75°N over a one-year period. Occurrence frequencies for each aerosol type are indicated. (**C**) Pixel-level intercomparison of POLDER/GRASP and MODIS/AQUA Dark Target (DT) AOD (550 nm) over ocean for coarse-mode conditions (POLDER AE<0.75); (**D**) Pixel-level intercomparison of POLDER/GRASP and MODIS/AQUA DT AOD (550 nm) over ocean with for fine-mode conditions (POLDER AE≥0.75).

Table S1. Validation of POLDER/GRASP and MODIS/AQUA DT+DB AOD (550 nm) against AERONET across land surface classes. Summary of validation metrics (RMSE and fraction within GCOS requirement) for POLDER/GRASP and MODIS/AQUA DT+DB AOD (550 nm) over four NDVI-based surface classes ($0 < NDVI \leq 0.2$, $0.2 < NDVI \leq 0.4$, $0.4 < NDVI \leq 0.6$, and $0.6 < NDVI \leq 1$). Normalization factors for each surface class are reported, with normalized metrics shown in brackets.

Land Cover		NDVI (0, 0.2]	NDVI (0.2, 0.4]	NDVI (0.4, 0.6]	NDVI (0.6, 1.0]	All NDVIs (Normalized)
Normalization Factors		0.343	0.202	0.197	0.258	-
POLDER 2008	RMSE	0.094	0.102	0.105	0.122	0.106 (0.105)
	GCOS (%)	47.9	51.8	52.2	57.9	52.8 (52.1)
MODIS 2008	RMSE	0.166	0.130	0.105	0.080	0.122 (0.125)
	GCOS (%)	33.6	46.1	49.6	59.5	47.9 (46.0)
MODIS 2018	RMSE	0.169	0.131	0.099	0.081	0.115 (0.125)
	GCOS (%)	30.9	47.2	52.8	60.8	50.6 (46.2)

Table S2. Validation of POLDER/GRASP and MODIS/AQUA DT+DB AOD (550 nm) against AERONET under fine- and coarse-mode aerosol regimes (coarse-mode aerosol: AE<0.75 and fine-mode aerosol: AE≥ 0.75). Summary of validation metrics (RMSE and fraction within GCOS requirement) for POLDER/GRASP and MODIS/AQUA DT+DB AOD (550 nm), stratified by dominant aerosol mode (coarse mode: AE<0.75; fine mode: AE≥ 0.75). Normalization factors for each aerosol size regime are reported, with normalized metrics shown in brackets.

Aerosol size regimes		Coarse-mode AE<0.75	Fine-mode AE≥ 0.75	All aerosol sizes (Normalized)
Normalization Factors		0.510	0.490	-
POLDER 2008	RMSE	0.121	0.101	0.106 (0.111)
	GCOS (%)	43.0	56.5	52.8 (49.6)
MODIS 2008	RMSE	0.150	0.110	0.122 (0.130)
	GCOS (%)	39.9	50.8	47.9 (45.2)
MODIS 2018	RMSE	0.142	0.113	0.115 (0.128)
	GCOS (%)	41.4	51.8	50.6 (46.5)
